# Engaging diverse communities participating in clinical trials: case examples from across Africa

**DOI:** 10.1186/1475-2875-9-86

**Published:** 2010-03-26

**Authors:** Aceme Nyika, Roma Chilengi, Deus Ishengoma, Sally Mtenga, Mahamadou A Thera, Mahamadou S Sissoko, John Lusingu, Alfred B Tiono, Ogobara Doumbo, Sodiomon B Sirima, Martha Lemnge, Wen L Kilama

**Affiliations:** 1 African Malaria Network Trust (AMANET), P. O. Box 33207, Dar es Salaam, Tanzania; 2 KEMRI-Wellcome Trust Research Programme, Kilifi District Hospital Grounds, P.O. Box 230, Kilifi, Kenya; 3 National Institute for Medical Research, Tanga Medical Research Centre, P.O Box 5004, Tanga, Tanzania; 4 Ifakara Health Institute (IHI), P. O. Box 78373, Dar es Salaam, Tanzania; 5 Malaria Research and Training Centre (MRTC), PB 1805, University of Bamako, Mali; 6 Centre National de Recherche et de Formation sur le Paludisme (CNRFP), 01 BP 2208 Ouagadougou 01, Burkina Faso

## Abstract

**Background:**

In the advent of increasing international collaborative research involving participants drawn from populations with diverse cultural backgrounds, community engagement becomes very critical for the smooth conduction of the research. The African Malaria Network Trust (AMANET) is a pan-African non-governmental organization that sponsors and technically supports malaria vaccine trials in various African countries.

**Case description:**

AMANET sponsored phase Ib or IIb clinical trials of several malaria vaccine candidates in various Africa countries. In Burkina Faso, Mali and Tanzania trials of the merozoite surface protein 3 -- in its Long Synthetic Peptide configuration (MSP3 LSP) -- were conducted. In Mali, the apical membrane antigen 1 (AMA1) was tested, while a hybrid of glutamate rich protein (GLURP) and MSP3 (GMZ2) was tested in Gabon. AMANET recognizes the importance of engaging with the communities from which trial participants are drawn, hence community engagement was given priority in all project activities conducted in the various countries.

**Discussion and evaluation:**

Existing local social systems were used to engage the communities from which clinical trial participants were drawn. This article focuses on community engagement activities employed at various AMANET-supported clinical trial sites in different countries, highlighting subtle differences in the approaches used. The paper also gives some general pros and cons of community engagement.

**Conclusions:**

Community engagement enables two-way sharing of accurate information and ideas between researchers and researched communities, which helps to create an environment conducive to smooth research activities with enhanced sense of research ownership by the communities.

## Background

### Conceptual framework of community engagement

Community engagement refers to the process of establishing two-way communication between researchers and the researched communities. There are many ways of engaging with communities due to different cultural, traditional, religious, or socioeconomic factors prevailing in the communities of interest. Efforts to ensure that participating communities understand the purpose and procedures of research could help to enhance mutual trust and to create a sense of collective ownership of research [1-4]. Exclusion of ordinary members of communities from which participants are drawn, over and above local beliefs and cultural practices, could create conditions that are conducive to the generation of misconceptions, rumours and suspicions about particular research projects, which could deter potential participants from taking part in the research or could hinder the progress of the research. The threat of negative consequential effects of such social roadblocks or bottlenecks on medical research and the potential involvement of the media, some pressure groups or politicians, imply that precautions should be taken to prevent or preemptively act to minimize the potential occurrence of such roadblocks or bottlenecks [5].

Such threats to the continuation of medical research could outweigh approvals given by the responsible Ministries of Health, ethics committees or regulatory authorities, as well as informed consent given by participants. One real life example of such scenarios is the case of the tenofovir multi-centre trials that were stopped in Cambodia, Cameroon and Thailand due to pressure from activist groups and the media [6]. Complaints raised against the Cambodian trial were lack of post-trial insurance, inadequate care for participants who became HIV positive during the trial and lack of community involvement in the planning of the trial. As for the Cameroonian trial, the complaints cited were inadequate informed consent and education regarding prevention of HIV. In Thailand, groups opposed to the Thai tenofovir trial cited ethical flaws in the trial design and lack of community involvement. It could be argued that all the problems encountered in these trials could have been prevented by involving the communities right from the beginning, instead of only engaging with other stakeholders such as ethics committees and local collaborating researchers who were probably considered to provide adequate community representation from the researchers' point of view and not from the participating communities' point of view. If their representation had been acceptable to the participating communities, the communities would probably not have felt sidelined. Thus the social dimension of health research requires engagement of the participating communities at the grassroots level.

For epidemiological genetic studies, community sensitization may be required to address potentially stigmatizing findings of the research. For instance, after several genetic studies showed that the BRCA1 and BRCA2 mutations, which are associated with a predisposition to breast cancer, and the APCI1307k allele associated with predisposition to colorectal cancer were found at higher rates in Ashkenazi Jews than the general population, Jewish people became concerned about possibility of stigmatization of Jews [7-9].

The increasing awareness of the need for sponsors and researchers to be sensitive to non-biomedical social aspects of health research and the increasingly cross-cultural nature of health research have contributed towards making health research more multi-disciplinary. For instance, the International HapMap Consortium, made up of partners from Canada, China, Japan, Nigeria and the UK, set up groups of partners with responsibilities to focus on scientific, analytical, legal, ethical and social issues of the project [10]. The HapMap consortium planned and budgeted for community engagement activities that were implemented before collection of samples and continued after the samples were collected. The activities included interviews, town meetings, focus group discussions, community survey and setting up of a Community Advisory Board (CAB) [11]. The CAB continued to receive funding annually after the life span of the consortium project in order to enable it to convene community meetings aimed at updating and consulting ordinary members of the communities.

It is, therefore, becoming increasingly imperative that social, legal and ethical issues surrounding health research be dealt with effectively and proactively in order to prevent them from becoming serious obstacles for the research. Community engagement helps not only to identify the pertinent issues, but also enables the communities concerned to contribute towards efforts to address the challenges. Such a participatory approach goes a long way in promoting mutual trust between researchers and communities.

### International guidelines on community engagement

Various international guidelines have also highlighted the importance of community engagement over and above individualized informed consent. For instance, CIOMS guideline 4 states that researchers are obliged to obtain informed consent from participants, and the commentary on the guideline stresses that "Sponsors and investigators should develop culturally appropriate ways to communicate information that is necessary for adherence to the standard required in the informed consent process" [12]. This requirement is explicitly stated in the 2006 supplement to the 2002 CIOMS guidelines where commentary on guideline 4 states that "The opinions of persons in a position equivalent to those whose biological samples or records would be used in a study offer a relevant point for determining whether such a study would offend community norms of privacy and autonomy. Such efforts are not the same as obtaining permission from community leaders to undertake a study..." [13].

The Nuffield Council on Bioethics (NCOB) 2002 report also stresses the importance of community involvement or assent when conducting research in developing countries. Paragraph 6.19 of the NCOB 2002 report states that "In some societies, it would be considered culturally inappropriate for researchers to ask individuals to participate in research without consulting the community or permission from community leaders" [14]. Paragraph 6.20 goes on to warn that "....to seek consent from an individual without seeking assent from leader(s) of the community, or creating public acceptance of research, may be considered disrespectful and may harm the relationships within that community and between a community and researchers". Thus, community engagement is emerging as a requirement for community-based research, in addition to ethical approval and informed consent.

### Responsibilities of sponsors and researchers regarding community engagement

Sponsors and researchers should, therefore, make efforts to enhance two-way communication with communities to discuss issues that may be of concern to the researched communities. Such communication could go a long way in fostering partnerships between researchers and communities based on mutual trust and a sense of collective ownership of the research. Partnerships could minimize risks of potential misunderstandings that could be fuelled by various factors such as rumours circulating in the communities, incorrect media reports that may crop up, and inadequate information and explanations given to participants.

It has been acknowledged that social value of research is as important as scientific validity [15]. The extra time and expense spent on community engagement and consultation processes help to add some value to the research by creating an atmosphere of openness and public trust because the processes involve (i) providing accurate, complete and balanced information to ordinary members of the community, (ii) giving ordinary people an opportunity to express their views, and (iii) taking into account and responding to communities' views wherever possible. Relying solely on approvals from local ethics committees and regulatory authorities and views from high-level technical advisory bodies without any practical efforts to interact directly with ordinary communities may in the long run prove to be unsatisfactory to the communities concerned and other stakeholders.

### African Malaria Network Trust project activities in Africa

The African Malaria Network Trust (AMANET) [16] is a pan-African non-governmental organization formed in 2002 from the African Malaria Vaccine Testing Network (AMVTN), which was founded in 1995. In an effort to promote conduction of health research in Africa that meets international scientific and ethical standards, AMANET takes a holistic approach that addresses infrastructural, human and financial resource needs of African research institutions. Consequently, AMANET implements a range of activities across Africa that range from capacity building of institutions and researchers to sponsorship of clinical trials of malaria candidate vaccines. AMANET recognizes communities from which research participants are drawn as critical stakeholders who should be engaged and treated as ends in themselves and not as mere means to an end. This paper outlines some examples of methods of engaging with communities that have been used in various parts of Africa where AMANET-sponsored clinical trials have been conducted. Some potential pros and cons of community engagement are also discussed.

Case description: practical examples of community engagement from Africa

### Burkina Faso

In Burkina Faso, AMANET sponsored two phase Ib trials of the merozoite surface protein 3 - in the long synthetic peptide configuration (MSP3-LSP) malaria vaccine candidate in adults [17] and in children [18]. There was no formal regulatory authority in Burkina Faso and the Ministry of Heath had the mandate to grant approval for clinical trials following favourable review outcome by the national ethics committee. The clinical trial site in Burkina Faso was at Balonghin, which is a rural village about 45 km from the capital city, Ouagadougou. The local study team had been doing research with this community for at least eight years. The researchers contributed substantially to the medical infrastructure and health care publicly available to the community, which enhanced community participation and cooperation in the local research projects.

**Figure 1:**
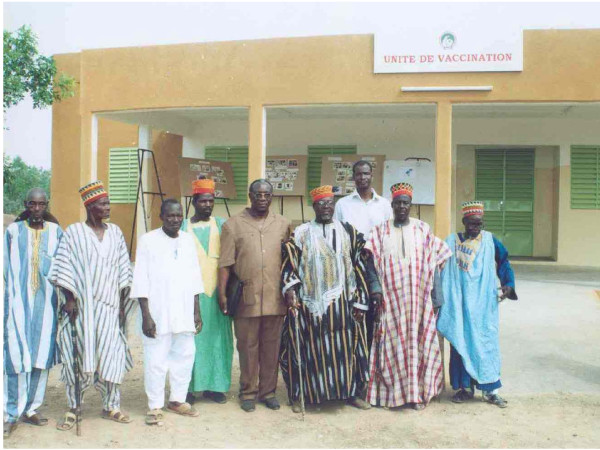
**Picture showing the researchers with community leaders after a meeting in Burkina Faso**.

In preparation for the clinical trials, several meetings with the communities were scheduled. The initial meetings were exclusively between the senior investigators and village chief with his elders. At these initial meetings, information about objectives, methodology, potential risks/benefits and importance of anticipated findings of the intended clinical trials was provided. Figure 1 shows some researchers with community leaders. Later, village meetings were held in smaller units explaining details of the proposed study by the research teams, taking time to answer their questions. The fact that the main potential benefit would be knowledge that could pave the way for further late phase trials was thoroughly explained. Repeated meetings were held in this manner until a sense of reasonable understanding and acceptance of the proposed study was reached. After the several meetings to explain the study, the village chief finally gave permission at a ceremonial meeting attended by villagers and other stakeholders. The big ceremonial meeting was marked with celebrations, food and traditional dances.

The experiences during the adult trial of MSP3-LSP malaria vaccine in Balonghin demonstrated that community engagement can be taken further to help in the random selection of participants. Briefly, the study was designed to include 30 adult participants randomised to receive either the study vaccine or control vaccine. In order to have a group of eligible adults, all men aged 18-45 years were invited for screening. A total of 112 volunteers were found to be eligible. A local traditional game was then employed at a gathering of the volunteers and other villagers to help transparently and randomly select those who would undergo the screening process for the study. The traditional game involved putting 52 short and 60 long sticks in a drum, the short sticks representing exclusion from the screening process of the study, while the longer sticks represented inclusion in the screening stage of the study. Each of the 112 volunteers had to blindly pick a stick from the drum without attempting to feel the length of the stick and in full view of cheering community members. The 60 villagers who picked the long sticks became the volunteers who proceeded to the screening stage aimed at selecting 30 trial participants. Therefore, the traditional game was successfully used to randomly and fairly select individuals to be screened with everybody attending the community meeting understanding the basis of selection as being equal chance for all the eligible candidates.

### Mali

In Mali an AMANET-sponsored phase Ib trial of the apical membrane antigen 1 (AMA1) malaria candidate vaccine, which could potentially prevent Plasmodium falciparum from invading red blood cells, was started in adults in 2007 [19,20]. Another AMANET-sponsored trial conducted in Mali was a phase IIb trial of the MSP3-LSP malaria vaccine candidate in children under the age of five years of age [21]; the MSP3-LSP vaccine had been shown to be acceptably safe in the trial in adults conducted in Burkina Faso. These clinical trials were conducted by the Malaria Research and Training Centre (MRTC), University of Bamako [22].

The experience of engaging with ordinary members of communities in Mali was gained from two different clinical trial sites; Bandiagara, a rural setting in the Dogon region, and Sotuba, a peri-urban settlement on the outskirts of the capital city, Bamako. The experiences at both field sites were essentially identical. Mali is generally a religious Muslim community with clearly structured administrative and traditional hierarchies. Both structures had to be respected in the approach to the potential study participants.

The first preparatory steps that had to be taken were administrative; firstly the Ministry of Public Health had to be officially informed, followed by the National Director of health, then the Regional Director of health, then the District Director of health, the District Prefect, and finally the Mayor. After the administrative steps, the process of community engagement was then initiated. To enter the research community, prior appointments had to be made with the chief. On the day of the appointment, the investigator team was led by the head of the local clinical site to the chief's residence where the village leaders, ordinary community members and local government officials gathered.

After systematic introductions and welcoming remarks by the village leaders and the chief, the research team introduced the subject of the new proposed study pointing out the type of the study, aims of the study and some information about collaboration between the local site and AMANET as sponsor of the trial. The chief's translator was translating into the local vernacular language. The community members and the chief asked questions throughout the whole meeting. Notably, contrary to expectations of typical African traditional settings, women were able to express their views freely. Since the trials were phase Ib or IIb, it was explained that even if the findings of the trials were positive, the investigational products would not be ready after the early phase trials but would need to undergo further studies before they can be licensed for use.

Following the discussions and responses to questions from the gathering, the chief spoke in support of the intended clinical trial and gave permission for the trial to be conducted. With the permission, research team and the community leaders organized a meeting with the ordinary members of the community that included heads of families. Any questions were allowed and answered by the research team. The engagement of the ordinary members of the community was done before, during and after the trial in order to ensure maintenance of high level of community trust and commitment. With an effective stepwise model of community engagement in place, the MRTC has conducted a total of ten phase I or II malaria vaccine trials with a follow-up rate of at least 95%. The approach established has also been used in other medical studies in Mali [2].

### Gabon

In Gabon, AMANET sponsored two phase Ib trials of GMZ2 malaria vaccine candidate, firstly in adults and secondly in children aged one to five years [23]. The GMZ2 vaccine is a promising candidate vaccine composed of two components, the glutamate rich protein (GLURP) and MSP3. The trial conducted in adults was aimed at assessing the safety of the vaccine before testing it in children. At the time of seeking approvals for the first study, Gabon neither had a national ethical committee nor a formal regulatory authority. A Regional ethics Committee called CERIL provided the ethical review and approval. After ethical approval was granted, the protocol and the approval were then submitted to the Ministry of Health, which played the role of the regulatory authority. Eventually, the ethical and regulatory approvals were used as supporting documents for the issuance of import permit to enable the importation of the candidate vaccine into Gabon.

Having obtained the necessary approvals and import permit, the researchers then arranged several community entry meetings starting with the village chief, and then village leaders. Upon permission being granted, the researchers organized several community meetings with the help of the community leaders. Announcements on the local radios and flyers were used to advertise the community meetings. Heads of families and their family members attended the general community meetings. In order to facilitate explanations to the members of the community, the researchers organized drama groups that conveyed information about the intended trial in vernacular language. The local collaborating researchers played a major role in packaging the information appropriately and in organizing the drama groups. During the meetings the researchers invited people to consider participating in the clinical trial if they met the inclusion criteria, which were explained. After the community engagement activities, which served to inform, sensitize and invite potential participants, recruitment commenced at the trial site within the district hospital. The informed consent process turned out to be very interactive and effective because most of the volunteers had attended the community meetings and were already aware of the pertinent issues that needed to be discussed.

### Tanga, Tanzania

NIMR Tanga Centre [24] received funding from AMANET in 2005 to prepare a site for malaria vaccine trial in Korogwe district, north-eastern Tanzania [25]. Demographic surveillance system (DSS) was established in 14 villages while passive case detection (PCD) of malaria fevers by Community Owned Resource Persons (CORPs) was done in four of the villages. In order to ensure that community members had a sense of ownership of the project activities, DSS workers and CORPs were recruited from the villages where the project was being conducted. Two malariometric surveys were conducted each year; during short (November/December) and long (May/June) rain seasons and these were preceded by village meetings to give details of the planned activities and to seek for community assent. For the surveys which were conducted after the baseline survey, the community members and district authority were given feedback of previous survey results and progress of DSS, and PCD of fevers.

After completion of the preparatory project, a phase 1b MSP3-LSP malaria candidate vaccine trial sponsored by AMANET was conducted in children under the aged 12 to 24 months [26]. The trial benefited from community engagement activities that had been carried out during the preparatory phase. Formal approvals were obtained from the national ethics review committee and the Tanzania Food and Drugs Authority (TFDA). Courtesy calls were then made to the district administrative officials. Regular contacts were made with the District Medical Officer (DMO) updating him at each stage of the preparations for the study. The DMO or representatives from his office accompanied the team when conducting some of the preparatory meetings.

At the village level, the first meeting was held with village leaders to explain the intended trial and seek permission to enter the village. The second meeting involved village elders and heads of families in addition to the village leaders. At this meeting information about the objectives of the trial, potential risks, envisaged benefits and procedures to be involved during the trial were explained. Approval to convene a third meeting for the villagers was sought and was granted. The third meeting was then held in order to inform the ordinary members of the community about the study. The aim, potential risks, potential benefits and procedures of the study were once again explained. Participants had an opportunity to ask questions which were adequately answered by the team of investigators. After the meeting, a copy of information sheet was given to parents/guardians of potential study participants to carry home and discuss with their families and later on ask more questions if any arose.

Following the various meetings, house-to-house sensitization which involved parents and other family members was done by the field workers. The field workers visited every household with children in the target age group to clarify issues about the study, which may have arisen since the village meeting, answering questions and clearing up misconceptions. The field workers were also responsible for verifying that children whose names were generated from the census list exist and to confirm their reported age using the Maternal and Child Health cards. In addition, the field workers identified other children in the eligible age range within the same household who might not have been registered during the census survey. Lastly, they invited potential study participants to attend screening sessions. The village had no local clinic or dispensary infrastructure sufficient to support the requirements of the trial, which was a randomized and blinded study. Thus, the local community donated a piece of land and the project constructed a modern facility for the trial and handed it over to the community for public health use as a local clinic after successful completion of the trial. The established model of community engagement was subsequently used during Phase IIb RTS,S malaria vaccine trial in Korogwe, Tanzania [27].

### Bagamoyo, Tanzania

Having realised the important role that Ethics Review Committees (ERCs) could play as a bridge between researchers and researched communities, the Ifakara Health Institute (IHI) ERC, using a sub-grant from AMANET, started a two-way community engagement programme aimed at conveying information about health research ethics in general to the communities in Bagamoyo, Tanzania. The Ifakara Health Institute [28] was formerly named Ifakara Health Research & Development Centre (IHRDC). In turn, the ERC and researchers enhanced their understanding of community concerns and perceptions of health research. Many clinical trials and other studies funded by various organizations draw participants from the communities in Bagamoyo [29,30]. For instance, Ifakara Health Institute has been recruiting participants from communities in Bagamoyo for the RTS,S multicentre trials being conducted in many African countries [31,32].

**Figure 2:**
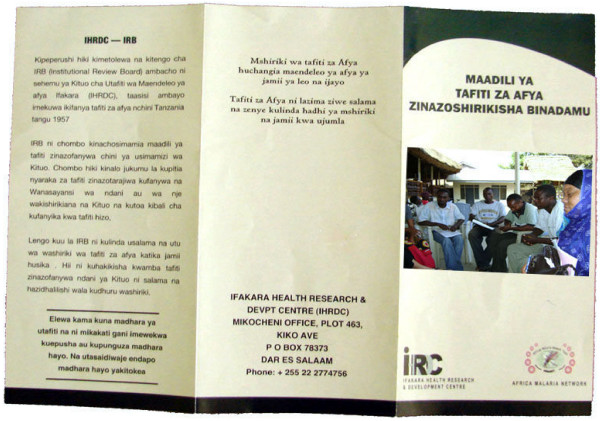
**Pamphlet and picture of community engagement activity by IHI IRB**.

**Figure 3:**
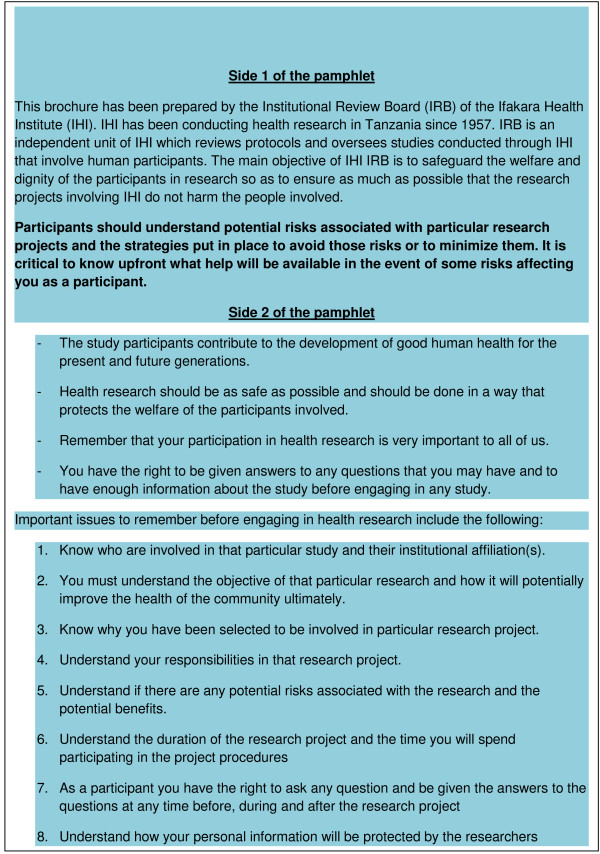
**Translation of the information on the pamphlet used by Ifakara Health Institute IRB**.

The IHI ERC firstly obtained permission from the community leaders to enter into their respective communities. Thereafter, the ERC organized focus group discussions with members of the communities in order to discuss issues considered to be critical for the conduction of ethical research and to understand the views or concerns of the ordinary members of the communities. Pamphlets that capture the important issues were then developed and subsequently used in discussions with the wider ordinary community members during community meetings. The pamphlets were also made freely available to the communities for possible further discussions with family members in their homes with the assistance of those who are literate. The pamphlets basically explained the difference between research and treatment, and the meaning of informed consent, potential risks and potential benefits in general terms. The identified issues summarized in the pamphlets (Figure 2) were thus used as a basis for further community engagement activities. Since the pamphlets were in the vernacular language, Kiswahili, members of the communities were able to discuss the issues among themselves, thus facilitating sensitization of the communities through dissemination of the information. Figure 3 gives the issues that are summarized in the pamphlets in English for the benefit of the readers.

In addition, members of a Community Advisory Board for IHI Bagamoyo Research Center were trained on basic health research ethics (HRE). Field researchers who work for Rufiji Demographic Surveillance System were also trained on basic HRE. This approach by the ERC of engaging communities to complement efforts that may be made by researchers could go a long way in encouraging participatory approach in health research so as to ensure that participants and their communities are treated as stakeholders who should be kept informed about research conducted on them.

## Discussion and evaluation

The experience gained in various African countries shows that community leaders, who are the gatekeepers for their communities, have to grant permission for researchers to enter into the community and conduct research. However, individual informed consent still had to be obtained from participants recruited into particular studies. The community leaders and the researchers explained that permission to enter the community was not meant to substitute individual informed consent. The permission given by the community leaders and heads of families should therefore not be considered to be the so called 'community consent', a term which could imply that individual informed consent is not necessary once permission has been granted by the community leaders.

**Figure 4:**
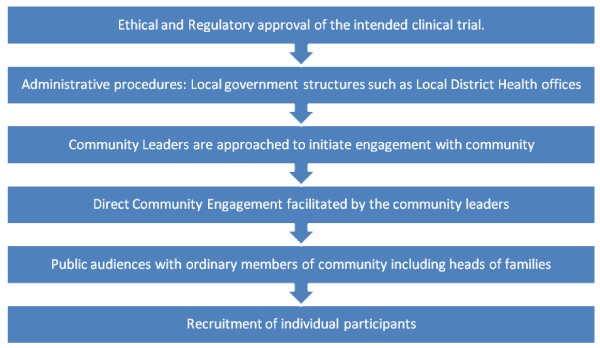
**General steps in community engagement at the various sites**.

Although the approach used in the different countries was basically the same (Figure 4), starting with administrative approval of the project, followed by permission to enter into the communities and finally meeting with the ordinary members of the communities, there were subtle procedural differences from site to site. Such differences were due to cultural and traditional differences in the social systems of the communities. However, these experiences showed that smooth implementation of any studies in these communities may not be possible if such site-specific community-wide and structured engagement is not followed. It is perhaps a distinctive contextual feature among African communities that mass mobilization with communal views on such issues as research within their community remains important.

The situation may be different in urbanized areas where households tend to mind their own family affairs following after the 'westernized cultural model'. In such urban settings, different approaches, which are feasible, could be used. It may be difficult to define a specific target community in such situations, and the term 'public engagement' may be more appropriate than 'community engagement'. For instance, researchers could explore using the media (print, radio and TV) to disseminate accurate information and to capture some views of the public.

**Figure 5:**
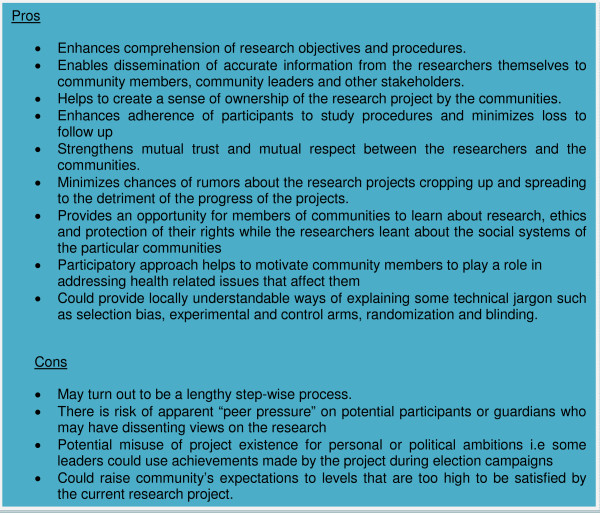
**Potential pros and cons of community engagement**.

In general, the process of community engagement basically shows respect for the communities that participate in research. Planning, setting aside time and budgeting for community engagement activities demonstrates that the communities are being treated as critical partners in the research process, and not as mere means for academic or commercial purposes. As outlined in Figure 5, community engagement helps to enhance comprehension of the research by the communities on one hand, and of community views and concerns by the researchers on the other. For instance, in Burkina Faso a local game that the community was familiar with was used to randomly select research participants. Thus although certain scientific jargon such as 'random selection' may not have equivalent terms in the vernacular language, the jargon could be made understandable if appropriate concepts or practices that may be available locally are used to elaborate the scientific procedures or jargon.

Some studies have shown that prospective participants in developing countries may need time to consult with their spouses or relatives before making a decision whether or not to participate in a research project [33,34]. Thus community engagement could go a long way in facilitating such internal family consultations since communities of the prospective participants may already be aware of the intended research projects. Overall, the community engagement process strengthens the relationship and trust between researchers and the communities.

However, community engagement also has some potential cons that should be watched out for and addressed whenever they arise. Firstly, although a timeframe may be put in place upfront, the process may take longer than planned due to the step-wise procedure, which is usually beyond the control of the researchers but is dependent on the existing local societal systems. Secondly, due to peer pressure, some individuals may feel obliged to take part in the proposed studies lest they are perceived as being against the wishes of the whole community, and may be treated as outcasts. Thirdly, the process of community engagement may raise the expectations of the community to levels beyond what the proposed study may be capable of tackling.

## Conclusions

The case examples from Africa show that involving communities from which research participants are drawn adds some social value to the research over and above the potential scientific benefits. Even if early phase trials do not yield products that may be used soon after completion of the trials, the communities appreciated the importance of such trials because the researchers made concerted efforts to explain and answer questions some of which may seem basic and trivial from a researchers point of view. The engagement with the communities created trust between the researchers and the communities, making the ordinary members of the communities feel a sense of ownership of the research.

Although there could be potential challenges such as lengthy processes and expectations that may be too high to meet, the overall potential benefits of engaging with target communities outweigh the challenges. Indeed, community engagement is an appropriate platform to address such challenges if they arise. The ordinary members of the communities do not necessarily have to understand the science behind the clinical trials, but need to understand such simple and straightforward issues as (i) the difference between research and health care delivery, (ii) purpose of doing the trial, (iii) research procedures involved such as number of injections and collection of blood samples, (iv) number of scheduled visits, (v) the known potential risks and (vi) the potential benefits such as knowledge about the safety and efficacy of the investigational product.

## Competing interests

The authors declare that they have no competing interests.

## Authors' contributions

AN made contributions to the project activities, gathered the case examples, drafted the paper, coordinated the inputs by co-authors and polished up the paper for submission as the corresponding author; RC made contributions to the project activities and made substantial intellectual inputs that helped to improve the draft; DI, SM, MAT, MSS, JL, ABT, OD, SBS, ML and WLK were involved in project activities at their respective sites and made intellectual contributions to the development of the paper. All authors approved the final version of the paper for publication.
